# Fluorescent knock-in mice to decipher the physiopathological role of G protein-coupled receptors

**DOI:** 10.3389/fphar.2014.00289

**Published:** 2015-01-06

**Authors:** Rhian A. Ceredig, Dominique Massotte

**Affiliations:** CNRS, Institut des Neurosciences Cellulaires et Intégratives, UPR 3212Strasbourg, France

**Keywords:** G protein-coupled receptors, fluorescent protein, knock-in, mouse model, drug design, biased agonism, receptor trafficking

## Abstract

G protein-coupled receptors (GPCRs) modulate most physiological functions but are also critically involved in numerous pathological states. Approximately a third of marketed drugs target GPCRs, which places this family of receptors in the main arena of pharmacological pre-clinical and clinical research. The complexity of GPCR function demands comprehensive appraisal in native environment to collect in-depth knowledge of receptor physiopathological roles and assess the potential of therapeutic molecules. Identifying neurons expressing endogenous GPCRs is therefore essential to locate them within functional circuits whereas GPCR visualization with subcellular resolution is required to get insight into agonist-induced trafficking. Both remain frequently poorly investigated because direct visualization of endogenous receptors is often hampered by the lack of appropriate tools. Also, monitoring intracellular trafficking requires real-time visualization to gather in-depth knowledge. In this context, knock-in mice expressing a fluorescent protein or a fluorescent version of a GPCR under the control of the endogenous promoter not only help to decipher neuroanatomical circuits but also enable real-time monitoring with subcellular resolution thus providing invaluable information on their trafficking in response to a physiological or a pharmacological challenge. This review will present the animal models and discuss their contribution to the understanding of the physiopathological role of GPCRs. We will also address the drawbacks associated with this methodological approach and browse future directions.

## INTRODUCTION

G protein-coupled-receptors (GPCRs) are proteins composed of seven transmembrane alpha helices with an extracellular *N*-terminus and an intracellular *C*-terminus (Rosenbaum et al., 2009). They represent one of the largest gene families in mammals and humans (Lagerström and Schiöth, 2008, and references therein). GPCRs can respond to various stimuli such as photons, ions, lipids, peptides, odorants, nucleotides, hormones, or neurotransmitters (Congreve et al., 2014). There are five human GPCR families: Rhodopsin, Secretin, Adhesion, Glutamate, and Frizzled/Taste2 with the rhodopsin receptor family being the largest. More than half of the 800 human GPCRs are classified as chemosensory taste or olfactory receptors (Lagerström and Schiöth, 2008; Heng et al., 2013). The remaining human GPCRs -roughly 370- may be involved in pathophysiological processes and are therefore potentially drugable targets. Indeed, metabolic, inflammatory, infectious or neurodegenerative diseases as well as cancer all involve a plethora of GPCRs (Heng et al., 2013). As many GPCRs belong to neuromodulatory systems (van den Pol, 2012), a large number of them are targeted by drugs in the context of nervous system disorders such as pain, drug addiction, anxiety, depression, sleep disorders, and neuroendocrine deregulation (Heng et al., 2013). Altogether, GPCRs represent the targets of about one third of marketed drugs (Overington et al., 2006).

Understanding the roles of GPCRs requires both in depth small scale investigation and overview. Indeed, GPCR expression, function, modulation, and trafficking properties remain difficult to fathom and reflect the complex, highly regulated pathways in which they are involved. The study of GPCRs in physiology and disease therefore requires integrative and functional systems. This is especially true when considering the central nervous system (CNS) where neuronal networks are complex and intermingled. It is therefore of utmost importance to identify and delineate cells that express the GPCR of interest. In the majority of studies, mapping GPCR expression was overcast by poor antibody specificity. The measure of this limitation was only fully appreciated when genetically modified mice which were deficient for the GPCR of interest became available, emphasizing the insufficient specificity of the commonly used antibodies, thereby prompting the search for new technologies to monitor receptor trafficking, decipher activated intracellular signaling cascades or investigate functional outcomes of GPCR activation in integrated systems, and particularly in neuronal networks (Marder, 2012). Among the options which were being explored, fluorescent proteins (FPs) isolated from natural organisms attracted special interest as they appeared to be very promising tools to achieve these goals. There are many advantages to using fluorescent molecular tags; the inherent fluorescence is directly visible, chemically resistant to fixation and can be used in time-course studies in living cells for tracking receptor trafficking events (Kallal and Benovic, 2000).

The Green FP (GFP) was the first FP used in biology. This protein is composed of 238 amino acids (roughly 27 kDa) and was isolated from the jellyfish *Aequorea victoria* (Shimomura et al., 1962, for review see Tsien, 1998). A mutant form of GFP called enhanced GFP (eGFP) was later generated, with improved quantum yield efficiency and higher solubility, making eGFP a popular reporter molecule (Cormack et al., 1997). The additional mutants that were created offer a large palette of fluorescence, ranging from violet to far red, thus opening new perspectives, including the possibility of co-expressing two or more FP in the same cell, whereby protein interactions could be investigated (Heim and Tsien, 1996). Likewise, this can be achieved by simultaneously expressing eGFP and mcherry, a stable monomeric mutant derived from the red fluorescent protein (RFP) DsRed, the latter was isolated from the coral *Discosoma sp.* (Campbell et al., 2002; Shaner et al., 2004). Additional variants derived from the GFP or DsRed were also generated and possess fast maturation, improved pH stability and photostability (reviewed in Shaner et al., 2007; Subach et al., 2009). The development of these FPs has been paralleled by technological advances in the field of live cell imaging that have brought high quality approaches for analysis of biological processes in a time- and space-dependent manner (Nienhaus and Nienhaus, 2014).

Validation of drug targets and pharmacological mechanisms cannot be achieved without *in vivo* preclinical studies for which mouse models provide a mammalian background and genetic tools of great value (Doyle et al., 2012; Bradley et al., 2014). In order to address GPCR function *in vivo,* tracking endogenous receptors with FPs therefore represents indisputable added value. In the following sections, we will review and comment on the use of FPs that has helped to shed light on endogenous GPCR function *in vivo.*

## *IN VIVO* EXPRESSION OF FP UNDER GPCR PROMOTER

### FROM TRANSGENIC TO KNOCK-IN MOUSE LINES

Transgenic mouse lines expressing FPs under the control of promoters for a GPCR or an endogenous peptide were created. A number of reporter mice generated using bacterial artificial chromosomes (BACs) were part of a project called gene expression nervous system atlas (GENSAT) http://www.gensat.org/index.html (Gong et al., 2003) that produced an important set of data relative to gene expression which could be used for deciphering the developmental implications and network dynamics of selected genes of interest. On the account that specific CNS genes are most often expressed in a particular cell population or anatomically defined structure, tandem dimer Tomato (td-Tomato), a RFP, or eGFP-labeling of these cells renders analysis of the anatomical, physiological and biomolecular properties of a chosen subtype of neurons accessible. Overall, transgenic reporter mouse lines have proven to be extremely useful for the precise mapping of GPCR and endogenous ligands expression in the nervous system, and are suitable for analysis of cell populations (Heintz, 2001).

The shortcomings of the transgenic mouse models are, however, manifold (Haruyama et al., 2009). (1) Transgenic expression results in overexpression compared to wild type animals. (2) Low efficiency of transmission to offspring may be caused by mosaic expression of the transgene in founder animals. Indeed, high copy number insertion of transgenes is more vulnerable to epigenetic silencing, which reduces the transgene expression level in successive generations. (3) Expression in unexpected tissues or timeframes may result from transgene insertion in genomic regions containing an endogenous promoter or enhancer. (4) Silencing or ectopic expression can be caused by positional effects. Transgene insertion can take place into transcriptionally inactive regions of the genome, or can be affected by neighboring repressor sites. Transgene insertion being, in essence, random, the possibility of disrupting the normal genome is very high. As a consequence, the erratic nature of the transgene insertion may result in unpredicted and/or detrimental phenotypes and off-target effects. As an example, many groups used BAC transgenic mice expressing eGFP driven by the promoter for either D_1_ or D_2_ receptors, the dopamine receptor 1 or 2, respectively (Lee et al., 2006; Bertran-Gonzalez et al., 2008; Valjent et al., 2009; Tian et al., 2010; Kramer et al., 2011; Chan et al., 2012). Mainly, work published using these two BAC transgenic mice successfully identified neurons expressing dopamine receptors and delineated dopaminergic connectivity in the CNS. However, Kramer et al. (2011) brought evidence of molecular and behavioral alterations in Drd2-eGFP BAC transgenic mice comprising novel environment hyperactivity, reduced locomotor response to cocaine, and D_2_ receptor agonist hypersensitivity. These effects were presumably due to unfortunate insertion of the BAC, which caused receptor overexpression (Kramer et al., 2011).

### KNOCK-IN MICE: TOWARD MORE SPECIFIC MODELS

**FIGURE 1:**
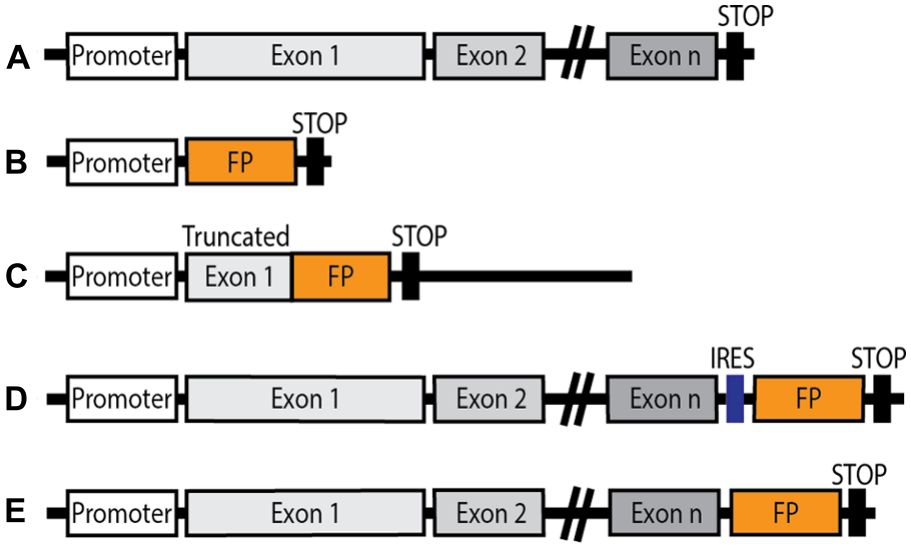
**Schematic diagram of genetic constructions of knock-in mice expressing a fluorescent protein (FP) under the control of an endogenous GPCR promoter. (A)** Endogenous GPCR gene layout. **(B)** Knock-in FP expressed under the control of the endogenous GPCR promoter: the endogenous GPCR gene is replaced by the FP coding sequence. **(C)** The FP coding sequence is knocked into the truncated gene coding for the native GPCR, resulting in genetic invalidation of the receptor. **(D)** Insertion of an internal ribosomal entry site (IRES) downstream of the endogenous GPCR gene, ahead of the FP coding sequence. Native GPCR expression is maintained, and the FP is also expressed under the control of the endogenous GPCR promoter. **(E)** The FP sequence is inserted in frame in place of the stop codon in the endogenous GPCR gene giving rise to a fluorescent fusion protein in which the FP is fused to the *C*-terminus of the functional GPCR in conditions of native expression.

To overcome the limitations associated with the use of transgenic mice, efforts were made to generate knock-in animals in which a FP is introduced at the locus of interest by homologous recombination. Several strategies are used (see **Figure 1**). Models in which an FP is expressed either under the control of an endogenous GPCR promoter are valuable and reliable tools for localization and characterization of cell population which express the GPCR of interest. However, such strategies present a significant drawback since the GPCR is non-functional following partial or total replacement of its coding sequence by the FP coding one. The FP is thus expressed in appropriate cells, but the precise subcellular localization and function of the receptor cannot be examined and the final outcome, in the case of homozygous animals, is the absence of the functional GPCR, equivalent to a knock-out phenotype. This limitation can be circumvented by the introduction of an internal ribosomal entry site (IRES) sequence, whereby expression of the endogenous GPCR is maintained and the chosen FP is expressed under control of the endogenous promoter.

#### Chemokine receptors

Jung et al. (2000) published the first knock-in mouse in which an FP was expressed under a GPCR promoter. The aim was to track cells which expressed the Fractalkin (CX_3_C) chemokine receptor CX_3_CR1, using a GFP knock-in strategy by replacing the first 390 bp of exon 2 of the *CX3CR1* gene that encodes the receptor *N*-terminus by a eGFP-coding sequence, enabling direct identification of peripheral blood cells and brain microglia expressing CX_3_CR1 (see **Table 1**). In heterozygous animals, CX_3_CR1 expression remained detectable because these CX_3_CR1^+/GFP^ heterozygous animals possess one allele for fluorescence visualization of cells expressing the GPCR of interest and one allele for expression of the functional receptor. Since CX_3_CR1 and its ligand Fractalkin play a role in immunological and inflammatory processes, this model was used to investigate microglia proliferation during early embryonic spinal cord invasion (Rigato et al., 2012) neuron-glia interactions in the context of nerve injury or neuroinflammation (Garcia et al., 2013) and in neurodegenerative diseases such as Alzheimer’s disease (Fuhrmann et al., 2010), or Parkinson’s disease (Virgone-Carlotta et al., 2013).

**Table 1:** Knock-in mice expressing fluorescent proteins under the control of G protein-coupled receptor (GPCR) endogenous promoters.

Targeted GPCR	Fluorescent protein	Identified cell type	Model	Therapeutic potential	Reference
**Insertion of FP sequence at the GPCR gene locus**
Chemokine CX_3_CR1	eGFP	Immune cells	Peritonitis Nerve injury	Neuroinflammation Neurodegenerative diseases	Jung et al. (2000)
		Microglia		Population dynamics in embryonic development	Rigato et al. (2012)
		Microglia	Neurodegeneration	Alzheimer	Fuhrmann et al. (2010)
		Microglia	Neuroinflammation	Parkinson	Virgone-Carlotta et al. (2013)
Chemokine CCR2	RFP	Immune cells	Experimental autoimmune encephalomyelitis	Neuroinflammation Neurodegenerative diseases	Saederup et al. (2010)
Chemokine CX_3_CR1 x Chemokine CCR2	eGFP RFP	Immune cells	Experimental autoimmune encephalomyelitis	Neuroinflammation Neurodegenerative diseases	Saederup et al. (2010)
		Myeloid cells Microglia	Experimental autoimmune encephalomyelitis	Population dynamics in embryonic development	Mizutani et al. (2012)
Oxytocin	Venus	Brain distribution	Anxiety related	Psychiatric disorders	Yoshimura et al. (2001)
		Spinal cord distribution		Nociception/pain	Wrobel et al. (2011)
Mrgprd	eGFPf	Sensory projections to epidermis		Nociception/pain	Zylka et al. (2005)
		Sensory projections to tooth pulp		Nociception/dental pain	Chung et al. (2012)
Taste TasR1	mcherry	Taste cells in taste buds and peripheral tissue		–	Voigt et al. (2012)
Taste Tas2R131	hrGFP	Taste cells in taste buds and peripheral tissue		–	Voigt et al. (2012)
Taste TasR1 x Taste Tas2R131	mcherry hrGFP	Taste cells in taste buds and peripheral tissue		–	Voigt et al. (2012)
**GPCR-IRES-FP expression**
Mas-related Mrgprd	eGFPf	Sensory projections to epidermis		Nociception/pain	Zylka et al. (2005)
Cannabinoid CB1	Td-Tomato	Neurons	Chronic cocaine injection	Drug addiction	Winters et al. (2012)

A follow-up to this knock-in mouse was published in 2010. In their paper, Saederup et al. (2010) designed a mouse with another single FP, RFP (a DsRed variant) replacing the first 279 base pairs of the open reading frame coding for the chemokine receptor type 2 (CCR2), and crossed the heterozygous CCR2^+/RFP^ and homozygous CCR2^RFP/RFP^ knock-in animals with the previously published CX_3_CR1^GFP/GFP^ homozygous animals, in order to obtain heterozygous double knock-in animals CX_3_CR1^+/GFP^CCR2^+/RFP^. The two chemokine receptors are expressed by distinct monocyte populations, therefore the red and green FPs constitute an elegant “two-colored” mouse model which was ideally suited for immunological studies (see **Table 1**). Indeed, because the immune system is constituted of cells that circulate in blood and lymph vessels, mature cells do not constitute a solid organ and are not restricted by connective tissue, therefore immune cell tracking is essential. Both the double heterozygous knock-in animals and the first mouse line (CX_3_CR1^+/GFP^ knock-in), were used to study and adequately quantify macrophage and monocyte population dynamics in a model of autoimmune tissue inflammation (experimental autoimmune encephalomyelitis), which recapitulates an animal model of multiple sclerosis (MS). In a subsequent study, the same group unveiled myeloid lineage and microglial chemokine receptor changes at embryonic stages 8.5–13.5, monitored CNS colonization by cells of interest, during development and in an MS model using adult mice (Mizutani et al., 2012). The knock-in models thus yielded exciting and fundamental results relative to the identification of cells expressing the designated GPCRs, and a fine description of cellular population changes in various disease paradigms.

#### Oxytocin receptors

Yoshida et al. (2009) engineered a mouse line in which a 5′ fragment of exon 3 of the oxytocin receptor (*OTR*) gene was replaced by a sequence coding for Venus FP, a yellow FP variant (Nagai et al., 2002). The recombined allele did not encode functional OTR but heterozygous animals retained radiolabelled oxytocin binding patterns through the intact allele, while enabling direct visualization of Venus in oxytocin expressing cells (Yoshida et al., 2009). Immunohistochemical analysis of brain sections from these animals revealed that there was a high expression of Venus (hence OTR) in monoaminergic areas of the brain in agreement with *in situ* hybridization (ISH) studies (Vaccari et al., 1998). However, the approach provided more sensitive detection of OTR expression by identifying additional areas and cells expressing Venus fluorescence among which serotoninergic ones. This study was the first to show evidence for interaction between oxytonergic and serotonergic systems in a pathway, which modulates anxiety. In a following study, these knock-in mice were used to map OTR expression in the spinal cord; shedding light on the modulatory role of oxytocinergic networks involved in spinal cord functions, such as nociception (Wrobel et al., 2011).

#### Taste receptors

Sensing of the chemical categories which are responsible for sweet, sour or umami taste is specifically encoded by GPCRs expressed on primary taste neurons (Liman et al., 2014). The taste receptor family 1 (Tas1r) belongs to class C GPCRs and function as obligatory heteromers, meaning that two GPCRs of different subtypes are associated and interact to form a functional entity. The taste receptor family 2 (Tas2r), on the other hand, are currently classified among class A GPCRs (Alexander, 2013).

In order to study the distribution of taste receptors in the mouse gustatory tissue, Voigt and collaborators engineered two knock-in mouse lines which they subsequently crossed in order to obtain double knock-in animals in which the open reading frame encoding the receptor was replaced by the sequence coding for the mcherry or humanized Renilla (hr)GFP under the control of Tas1r1 (umami taste receptor) or Tas2r131 (bitter taste receptor) promoters, respectively (Voigt et al., 2012). This approach permitted identification of cells expressing mcherry under the control of the Tas1r1 promoter in the lingual papillae, soft palate, fungiform and foliate papillae, confirming previous findings (Hoon et al., 1999; Stone et al., 2007) but also in extra-gustatory tissues (lung epithelium, testis, thymus) which had not been investigated before (Voigt et al., 2012). Expression of hrGFP under the control of Tas2r131 promoter was in accordance with previously findings describing taste receptor distributions (Behrens et al., 2007), showing abundant hrGFP expression in taste buds of the posterior tongue, vallate palate and foliate palate. In addition, it uncovered, for the first time, expression restricted to only half of the bitter sensor cells (Voigt et al., 2012). Double knock-in animals lacked both taste receptors, but expressed FPs in the targeted cells [verified by reverse transcription polymerase chain reaction (RT-PCR), ISH and immunohistochemistry]. This genetic labeling technique served for population distribution studies, which was until then unachievable, given the fact that Tasr expression is sparse in cells, and that the available antibodies lack specificity. The double knock-in animals yielded a valuable and detailed cartography of taste receptors in the mouse, and revealed that distinct chemosensory cell populations mediate specific and non-overlapping taste qualities.

#### Mas-related-G-protein coupled receptors

Mas-related-G-protein coupled receptor member D (Mrgprd) belongs to a GPCR family of approximately 50 members, related to *Mas1* (oncogene-like MAS), called Mrgs. Mrgs are suspected to be involved in development, regulation and function of nociceptive neurons or nociceptors (Dong et al., 2001) and are expressed in a subset of nociceptors, which are small diameter primary sensory neurons in dorsal root ganglia (DRG) directly involved in processing nociceptive stimuli, especially itch (Liu et al., 2012).

Zylka et al. (2005) observed similar expression patterns of the eGFPf (a farnesylated form that anchors the FP to the cytoplasmic leaflet of the lipid bilayer) in nociceptors, and projections of the sensory neurons to the epidermis using knock-in mice in which the open reading frame coding for Mrgprd is replaced by the sequence encoding the eGFPf or knock-in animals in which the eGFPf sequence is inserted behind an IRES element downstream of the mouse Mrgprd gene (Zylka et al., 2005). This demonstrates that both strategies can be equally used for cellular mapping. In addition, similar projection profiles in the epidermis validated the eGFPf knock-in mouse for axonal tracing by comparison with the widely used human placental alkaline phosphatase tethered to the extracellular surface of the plasma membrane by a glycophosphatidylinositol linkage.

In a later study, the knock-in mouse model expressing eGFPf at the Mrgprd locus was used to identify non-peptidic nociceptive neurons of trigeminal ganglia innervating tooth pulp (Chung et al., 2012). This opens future application of this model to study the role and function of the targeted GPCR in dental pain.

#### Cannabinoid receptors

The endocannabinoid system plays roles in memory, appetite, stress and immune processes, as well as motivation and emotional responses and modulates the effects of some drugs of abuse (Pertwee, 2006; Tan et al., 2014). In the nucleus accumbens (NAc), a brain structure which has a crucial role in reward processing and a decisive influence on emotional and motivational responses, cannabinoid receptor 1 (CB1) expression is limited but nevertheless essential for cocaine-induced reward in mice (Marsicano and Lutz, 1999). In order to further identify and delineate the cellular and electrophysiological properties of CB1 receptor expressing cells in the NAc, Winters et al. (2012) designed a knock-in mouse line in which an IRES element ensures expression of both CB1 receptors and td-Tomato under the control of the CB1 promoter. Importantly, this mouse line still expressed functional CB1 receptors. Neurons expressing CB1 receptors were readily visualized in the NAc and their distribution was in accordance with previous data on CB1 receptor localization using ISH or immunohistochemistry (Mailleux and Vanderhaeghen, 1992; Tsou et al., 1997). This mouse line enabled to identify of cells and to explicitly demonstrate biochemical and signaling properties of a particular neuronal population of fast-spiking interneurons in the NAc which impacts on the NAc projections and connectivity. Results also revealed functional impact of cocaine on these neurons (Winters et al., 2012).

## GPCR-FP FUSION FOR *IN VIVO* FUNCTIONAL AND MAPPING STUDIES

### INITIAL VALIDATION OF GPCR-FP FUSIONS IN HETEROLOGOUS SYSTEMS

Fusions between a GPCR and an FP as tools to monitor the GPCR subcellular localization and trafficking were first studied in heterologous systems. Two fusion options were considered: either the FP at the *N*-terminus or at the *C*-terminus. A vast majority of GPCRs do not have cleavable *N*-terminus signal sequences that target them to the plasma membrane. Introduction of a foreign sequence ahead of their *N*-terminus has been shown to disrupt surface addressing, and correct membrane targeting and insertion therefore requires introduction of an additional foreign signal sequence in front of the fusion construct (McDonald et al., 2007). If proper cell surface expression is indeed restored, introduction of such a signal sequence nonetheless strongly impacts on the relative ratio between surface expression and intracellular distribution by substantially increasing the amount of protein at the cell surface (Dunham and Hall, 2009, and references therein). Hence, such fusion proteins are not well suited to mimic the responses of endogenous GPCRs to agonist stimulation and were not used for *in vivo* studies.

Concerns have also been raised regarding in frame insertion of the FP at the *C*-terminus of the GPCR by substitution of the stop codon. The presence of a 27 kDa beta barrel at the intracellular extremity of the GPCR could indeed interfere with intracellular scaffold partners and modify signaling or internalization processes thus defeating the object when studying GPCR signaling properties. However, many studies performed in mammalian cells on a large number of GPCRs strongly suggest that addition of GFP at the *C*-terminus does not significantly affect subcellular distribution in the basal/unstimulated state, ligand binding or agonist-induced receptor phosphorylation and internalization, (for review Kallal and Benovic, 2000). McLean and Milligan (2000) expressed β_1_- and β_2_-adrenergic receptors fused to a *C*-terminal eGFP mutant in human embryonic kidney (HEK 293) cells. These authors concluded that the presence of the eGFP did not influence ligand binding but decreased the agonist-induced internalization kinetics without affecting the intracellular fate of the receptor. Trafficking of the fusion protein was qualitatively maintained, but was quantitatively slightly modified compared to native proteins. This study therefore supports the use of such fusions to monitor endogenous receptor subcellular localization. Similarly, the genetic construction encoding the delta opioid (DOP) receptor fused with eGFP protein at the *C*-terminus was expressed in transfected HEK 293 cells, and the fusion did not alter opioid ligand binding affinity or signaling (Scherrer et al., 2006). This construct was later successfully used to express a functional DOP-eGFP fusion in mice by knocking the modified sequence into the endogenous DOP receptor locus (Scherrer et al., 2006, see below).

In some cases, however, FP fusion at the GPCR C-terminus had deleterious effects. Defective targeting to the cell surface was reported for the melanocortin 2 receptor fused to the GFP in HEK 293 cells (Roy et al., 2007) and no recycling was observed for the muscarinic M4 receptor fused to a *C*-terminal red variant of GFP in neuroblastoma/glioma hybrid cells (NG108-15 cells; Madziva and Edwardson, 2001). In both cases, impairment was more likely to be due to gross overexpression rather than fusion of the FP to the *C*-terminus. High levels of expression of a GPCR in a non-native environment can indeed artificially elicit properties and interactions that would not occur *in vivo*. Moreover, cell lines used for heterologous expression may provide different intracellular machinery for complex protein folding or post-translational modifications compared to naturally producing cells. This represents an additional limitation to the study of GPCR functions and prompted to develop *in vivo* approaches.

### FROM TRANSGENIC TO KNOCK-IN MOUSE LINES

Papay et al. (2004) reported a transgenic mouse model of a fluorescent tagged GPCR. The construct they described was composed of a 3.4 kb fragment of the mouse endogenous α1B adrenoceptor promoter, the human α1B adrenoceptor coding sequence with *C*-terminal fusion eGFP sequence. The resulting founder lines were characterized, and high expression levels were observed in all tissues that naturally express α1B adrenoceptors by fluorescence microscopy. Binding affinities and internalization profiles were similar to those of endogenous receptors. With this study, Papay et al. (2004) reported the first mouse model expressing a GPCR tagged with eGFP as a transgenic approach for *in vivo* GPCR localization studies. The generation of knock-in animals represented a further improvement by enabling for the first time to track down endogenous receptors, which has opened a new era for pharmacological research.

### KNOCK-IN HUMANIZED RHODOPSIN FUSED WITH A FLUORESCENT PROTEIN (hRho-eGFP)

Chan et al. (2004) mouse lines expressing human rhodopsin-eGFP were engineered using different knock-in strategies. All mouse lines showed decreased expression levels of the fusion protein relative to the endogenous mouse rhodopsin. Comparing the different homozygote mouse lines enabled to correlate the decrease in human rhodopsin–eGFP expression to the increased rate of retinal degeneration, providing a model of human diseases. More recently, using a human mutant rhodopsin allele [proline-to-histidine change at codon 23 (P23H) rhodopsin] which induces mislocalization and degradation of the human protein, the research group generated a knock-in mouse line which modeled a common cause of autosomal dominant retinitis pigmentosa (Price et al., 2011). In humans, mutation Q344X is responsible for a severe early onset form of retinitis pigmentosa. The Q344X mutation introduces a premature stop codon that prevents GFP expression in the human rhodopsin-eGFP construct. Knock-in animals expressing this mutant construct were used to monitor eGFP fluorescence recovery as an index of the frequency and timing of somatic mutations in the rhodopsin gene (Sandoval et al., 2014). These mouse lines provided substantial and valuable data concerning rhodopsin distribution in the retina (for references, also see **Table 2**), and were advantageously implemented for non-invasive measurement by illuminating the mouse retina in live animals with blue light (Wensel et al., 2005). They will provide a means to assess the impact of future gene-targeting treatment strategies for retinal degeneration (Gross et al., 2006; Sandoval et al., 2014).

**Table 2:** Knock-in mice expressing GPCR-fluorescent protein fusions.

Fusion construct	Biological readout	Reference
hRhodopsin-eGFP	Retinal degeneration kinetics (model of recessive retinitis pigmentosa)	Chan et al. (2004)
	Distribution, membrane structure, and trafficking of rhodopsin (model of retinitis pigmentosa)	Gross et al. (2006)
P23H-hRhodopsin-eGFP	Degeneration and degradation kinetics of rhodopsin (model of common cause of autosomal dominant retinitis pigmentosa)	Price et al. (2011)
Q344X-hRhodopsin-eGFP	DNA repair in photoreceptors cells during retinogenesis (degeneration and degradation kinetics in a model of severe early-onset of retinitis pigmentosa)	Sandoval et al. (2014)
DOP-eGFP	Receptor distribution: –central nervous system	Scherrer et al. (2006; 2009), Erbs et al. (2014)
	– hippocampus	Erbs et al. (2012), Rezai et al. (2012, 2013)
	– dorsal root ganglia	Scherrer et al. (2009); Bardoni et al. (2014)
	– mechanosensors in the skin	Bardoni et al. (2014)
	– myenteric plexus	Poole et al. (2011)
	Correlation between behavioral desensitization and receptor internalization	Scherrer et al. (2006); Pradhan et al. (2009, 2010)
	Biased agonism at the receptor – pharmacological drugs – endogenous opioid peptides	Pradhan et al. (2009, 2010) Faget et al. (2012)
	Behaviorally controlled receptor subcellular distribution	Faget et al. (2012); Bertran-Gonzalez et al. (2013), Laurent et al. (2014)
MOP-mcherry	Receptor distribution in the central and peripheral nervous systems	Erbs et al. (2014)
MOP-mcherry x DOP-eGFP	MOP-DOP neuronal co-expression in the brain	Erbs et al. (2014)

### OPIOID RECEPTORS

The opioid system modulates a wide range of physiological states, of which nociception, reward, mood, stress, neuroendocrine physiology, immunity, autonomic functions such as gastro-intestinal transit (Kieffer and Evans, 2009; Walwyn et al., 2010; Chu Sin Chung and Kieffer, 2013; Lutz and Kieffer, 2013). Opioid receptors are members of the class A GPCR family, mu (MOP), delta (DOP) and kappa (KOP) opioid receptors couple to inhibitory heterotrimeric inhibitory G protein, and have high sequence homology (Akil et al., 1998).

#### Mapping of receptor expression with neuronal resolution

Scherrer et al. (2006) generated a DOP-eGFP knock-in mouse line by homologous recombination in which the coding sequence for the DOP receptor fused to its *C*-terminus to the eGFP was inserted at the *Oprd1* locus.

Delta opioid-eGFP knock-in mice proved very helpful to map DOP receptors in the nervous system and remedy the lack of highly specific antibodies (see **Table 2**). In the peripheral nervous system, DOP-eGFP receptors were detected in cell bodies of specific peripheral sensory neuronal populations which process sensory stimuli, namely mostly in large diameter myelinated (Neurofilament 200 positive), and in small diameter unmyelinated non-peptidergic (Isolectin B4 positive) neurons (Scherrer et al., 2009; Bardoni et al., 2014). The expression pattern of DOP-eGFP receptors was also reported in mechanosensory organs in the skin (Bardoni et al., 2014). Another study focused on the distribution of DOP-eGFP in enteric neurons with DOP-eGFP expression mainly in secretomotor neurons of the submucosal plexus of the digestive tract (Poole et al., 2011). The observed distribution reflects functional roles of DOP receptors in inhibition of intestinal motility and absorption.

In the CNS, DOP-eGFP mapping was performed in the brain and spinal cord (Erbs et al., 2014). Detailed DOP-eGFP expression was also reported in the hippocampus, where functional DOP-eGFP was found to be mainly expressed in GABAergic interneurons, mostly parvalbumin-positive ones (Erbs et al., 2012; Rezai et al., 2013). The DOP-eGFP knock-in mice also enabled to resolve the debate concerning the presence of DOP receptors in principal cells. The absence of colocalization with calbindin (Erbs et al., 2012) and presynaptic expression restricted to afferents to glutamatergic principal cells established that no functional DOP receptors are expressed under basal conditions in those cells (Rezai et al., 2012). These results are consistent with a modulation of principal cell activity in the hippocampus by DOP receptors, and therefore an impact of the receptors in learning and memory.

More recently, a knock-in mouse line expressing a MOP receptor fused with a RFP at the C-terminus, MOP-mcherry, was generated by Erbs et al. (2014). At the *Oprm1* locus, mcherry cDNA was introduced into exon 4 of the MOP gene in frame and 5′ from the stop codon. This FP is monomeric and highly photostable, and the strong red signal of MOP-mcherry fusion protein enabled direct identification of neurons expressing MOP in the nervous system (Erbs et al., 2014). The authors compiled the DOP-eGFP and MOP-mcherry distributions in a neuroanatomical atlas available at http://mordor.ics-mci.fr

Several studies in heterologous systems or cell culture had suggested that MOP and DOP receptors may interact to form heteromers (van Rijn et al., 2010; Rozenfeld et al., 2012; Stockton and Devi, 2012) but their existence *in vivo* remains debated. Co-immunoprecipitation studies performed on tissue from spinal cord or DRGs also hinted at close physical proximity between the two receptors in these areas (Gomes et al., 2004; Xie et al., 2009). In addition, MOP-DOP heteromers had been detected in some brain areas using specific antibodies (Gupta et al., 2010). Recently, extensive mapping of MOP-DOP neuronal colocalization using double knock-in mice co-expressing DOP-eGFP and MOP-mcherry provided sound data to investigate MOP-DOP physical proximity and functional interactions. In the hippocampus, a brain area where the two receptors are highly co-expressed, co-immunoprecipitation experiments using antibodies raised against the FPs indeed confirmed physical proximity (Erbs et al., 2014). These animals will now be useful to address MOP-DOP specificities in ligand binding, signaling and trafficking as well as functional output and to investigate the potential of MOP-DOP heteromers as a novel therapeutic target.

#### *In vivo* trafficking, desensitization and behavioral output

The DOP-eGFP mouse line is the first example of the use of a knock-in line to study GPCR functions *in vivo* (Scherrer et al., 2006). DOP agonist-induced internalization was observed *in vivo* upon activation by the alkaloid [(+)-4-[(alphaR)-alpha-((2S,5R)-4-allyl-2,5- dimethyl-1-piperazinyl)-3-meth oxybenzyl]-N,*N*-diethylbenzamide] (SNC-80) and the endogenous peptide Met-enkephalin (Scherrer et al., 2006). The two agonists induce receptor internalization in heterologous systems with receptor phosphorylation as the first step of a cascade of events leading to termination of G protein dependent signaling, receptor removal from the cell membrane and trafficking to intracellular compartments (Ferguson et al., 1996; von Zastrow and Williams, 2012; Walther and Ferguson, 2013). DOP-eGFP mice revealed that these agonists also induce receptor phosphorylation, internalization via clathrin coated pits *in vivo* and degradation in the lysosomal compartment in the brain (Scherrer et al., 2006; Pradhan et al., 2009; Faget et al., 2012) and peripheral nervous system in the myenteric plexus (Poole et al., 2011) and DRGs (Scherrer et al., 2009). Moreover, these animals prove to be instrumental to decipher molecular mechanisms underlying receptor desensitization leading to a loss of responsiveness of the receptor upon stimulation by an agonist. Scherrer et al. (2006) were indeed able, for the first time, to establish the correlation between receptor trafficking *in vivo* and the behavioral response: namely that the receptor internalization induced by acute administration of the agonist SNC-80 was responsible for the observed locomotor desensitization. This paper was followed by additional studies exploring the consequences of receptor pharmacological stimulation in more detail, in particular the concept of biased agonism.

G protein-coupled receptors have a flexible and highly dynamic nature (Moreira, 2014) which enables a given ligand to show functional selectivity, that is, preferential activation of signal transduction pathways, otherwise termed biased agonism (Ostrom and Insel, 2004; Giguere et al., 2014; Kenakin, 2014). DOP-eGFP mice offer the possibility of addressing this concept *in vivo* and to link it to a functional response. DOP-eGFP mice were used to analyze the properties of two DOP receptor agonists possessing similar signaling potencies and efficacies but with different internalization profiles (Pradhan et al., 2009). SNC-80 and N,*N*-diethyl-4-(phenyl-piperidin-4-ylidenemethyl)-benzamide (AR-M100390), with high and low internalization properties respectively, were systemically administered to mice, and receptor trafficking was correlated to induced anti-allodynic effect in the context of inflammatory pain (Pradhan et al., 2009). As expected, acute SNC-80 administration resulted in receptor phosphorylation, decreased G protein coupling and receptor degradation in the lysosomal compartment, leading to desensitization with loss of anti-allodynic properties. On the other hand, acute injection of AR-M100390 did not result in receptor phosphorylation, did not reduce G protein coupling, did not induce receptor internalization or desensitization but retained analgesic properties. This study demonstrated that DOP receptor localization determines its function *in vivo* and highlights the importance of receptor tracking in order to extricate behavioral and cellular correlates of specific agonist properties (Pradhan et al., 2009).

In a following study, DOP-eGFP mice were used to assess the physiological impact of distinct signaling pathway recruitment and/or adaptive responses upon chronic administration of two DOP receptor agonists (Pradhan et al., 2010). Chronic administration of SNC-80, which has high internalization properties, led to marked receptor downregulation and degradation in SNC-80-tolerant animals. Receptor internalization prevented any additional activation through physical disappearance from the cell surface leading to general desensitization, as assessed by thermal and mechanical analgesia, locomotor activity and anxiety-related behavior. On the other hand, chronic administration of AR-M100390, with weak internalization properties, did not cause changes in DOP-eGFP localization and induced tolerance restricted to analgesia, with no effect on locomotor activity or anxiolytic responses. These data show that a selective internalization-independent tolerance was elicited and suggest the occurrence of adaptative mechanisms that are network dependent. These findings reinforce the importance of understanding agonist specific signaling underlying biased agonism and tolerance. Considering that drug design has focused on offering orthosteric or allosteric modulators of GPCRs (Bradley et al., 2014), research groups need to explore the downstream signaling cascades of these drugs in more detail in order to understand and target the molecular events which underlie their efficacy. This is an essential progress for the understanding of drug action and opens new possibilities for drug design.

Direct visualization of the receptor also permitted to decipher the functional role of delta receptors in neuronal networks and to understand the complex relation between behavior and receptor subcellular distribution. Of particular interest is the observation that DOP subcellular distribution is modified in two brain areas involved in the processing of information associated with emotional value or predicted outcome. The CA1 area of the hippocampus is known to operate as a coincidence detector that reflects association of the context with strong emotional stimuli of positive or aversive value (Duncan et al., 2012). Accordingly, increased c-Fos immunoreactivity revealed activation of this region in a drug-context association paradigm, and DOP-eGFP internalization in this area therefore suggested a modulatory role of the receptor in behavioral responses linked to context-induced withdrawal (Faget et al., 2012). Along the same line, persistent increase of DOP-eGFP expression at the cell surface of cholinergic interneurons was induced by conditioned training in the NAc shell, which is involved in decision making and predictive reward evaluation upon pavlovian conditioning (Bertran-Gonzalez et al., 2013; Laurent et al., 2014).

Finally, the knock-in strategy revealed that the DOP-eGFP internalization profile in response to endogenous opioid release is distinct from what is observed upon pharmacological stimulation (Faget et al., 2012). Indeed, only part of the receptor population present at the cell surface underwent internalization under physiological conditions. This observation further highlights the need to take into account the extent of changes that drug administration induces in receptor cellular distribution.

#### Methodological improvements

Interestingly, DOP-eGFP knock in mice also bring useful technical insight. During the process of acute brain slice preparation for electrophysiological recordings, DOP-eGFP revealed spontaneous receptor internalization (Rezai et al., 2013). This event was likely due to high glutamatergic activity in the hippocampus upon slicing that leads to exitoxicity. Direct visualization of the receptor therefore revealed a bias associated with previously unrecognized receptor trafficking that can now be addressed by initiating optimization of slice preparation conditions for electrophysiological recording (Rezai et al., 2013). This observation may be of particular relevance when addressing cellular responses elicited by drug application.

## CONCERNS ABOUT THE USE *IN VIVO* OF GPCR-FP FUSIONS FOR FUNCTIONAL STUDIES

Despite the undeniably wide advances which have been and will be brought by genetically engineered mice encoding fluorescent endogenous GPCRs, concerns were raised regarding the inherent consequences of genetic manipulation. The possibility that the observed localization does not entirely reflect the wild type receptor distribution appears irrelevant since both MOP-mcherry and DOP-eGFP receptor distributions in the brain are in full agreement with reports in mice and rats based on ligand binding (Kitchen et al., 1997; Slowe et al., 1999; Goody et al., 2002; Lesscher et al., 2003), GTPγS incorporation (Tempel and Zukin, 1987; Pradhan and Clarke, 2005) or mRNA detection [George et al., 1994; Mansour et al., 1995; Cahill et al., 2001; for a review see (LeMerrer et al., 2009)]. Also, in a more detailed study, DOP-eGFP expression in the hippocampus, mainly in parvalbumin-positive GABAergic interneurons (Erbs et al., 2012), was corroborated by ISH studies on DOP receptors (Stumm et al., 2004).

In the peripheral nervous system, despite previous reports suggesting SP-dependent trafficking of DOP receptors to the cell membrane (Guan et al., 2005), Scherrer et al. (2009) reported that DOP-eGFP almost never co-localized with substance P (SP) in peripheral sensory neurons (Scherrer et al., 2009), a finding that was debated by others (Wang et al., 2010). A more recent study addressed this discrepancy by comparing DOP-eGFP cellular distribution to that of the native DOP receptor using an ultrasensitive and specific ISH technique, which can detect single mRNA molecules (Bardoni et al., 2014). Patterns of DOP-eGFP distribution and *Oprd1* mRNA expression were found to be very similar and detectable in the same neuronal populations, namely mostly in large diameter myelinated cells (Neurofilament 200 positive), and in small diameter unmyelinated non-peptidergic neurons (isolectin B4 positive; Bardoni et al., 2014). These data unambiguously confirm that the expression profile of the fluorescent constructs mimics the endogenous one and that fluorescent knock-in mice can be reliably used for mapping receptors in the central and peripheral nervous system.

Regarding functional aspects, there has been no evidence so far of any overt phenotypical or behavioral differences between the DOP receptor knock-in strain and wild type animals (Scherrer et al., 2006; Pradhan et al., 2009, 2010; Rezai et al., 2013), despite a twofold increase in mRNA and protein levels as well as increased G protein activation compared to wild type animals (Scherrer et al., 2006). However, the possibility that the subcellular distribution of the fluorescent fusion does not recapitulate that of the native untagged receptor is still debated. Indeed, high surface expression of DOP-eGFP is observed under basal conditions in several brain regions, particularly in the hippocampus (Scherrer et al., 2009; Erbs et al., 2012, 2014; Faget et al., 2012). This does not correlate with previous studies on wild type receptors using electron microscopy or fluorescent ligands that indicated a predominant intracellular localization under basal conditions and surface recruitment upon chronic morphine or chronic pain condition (Cahill et al., 2001; Morinville et al., 2004; Gendron et al., 2006; for review see Cahill et al., 2007; Gendron et al., 2014). Surface expression of DOP-eGFP, however, varies across CNS regions and neuronal type whereas high fluorescence is always visible within the cytoplasm (Erbs et al., 2014). Accordingly, high surface expression appears to be restricted to some neuronal types such as GABAergic interneurons in the hippocampus or large proprioceptors in DRGs (Scherrer et al., 2006; Erbs et al., 2014). In many areas where DOP receptors are highly expressed such as the striatum, the basal ganglia, the amygdala or the spinal cord, DOP-eGFP is not readily detected at the cell surface (Erbs et al., 2014) suggesting that DOP-eGFP intracellular localization is predominant in those neurons. Importantly, surface expression of DOP-eGFP can be augmented under physiological stimulation (Bertran-Gonzalez et al., 2013; Laurent et al., 2014; see above) or increased upon chronic morphine treatment as previously reported for wild type receptors (Erbs et al., unpublished data), strongly supporting that the fused FP does not impact on the native subcellular distribution of the receptor and that the latter can be modulated according to the physiological state or modified upon pharmacological treatment.

In the case of MOP-mcherry knock-in mice, the red fluorescent signal is stronger inside the cell than at the plasma membrane (Erbs et al., 2014). This distribution reflects actual receptor intracellular distribution, as evidenced by comparison with MOP-specific immunohistochemistry in heterozygous mice, which confirms that the fusion protein does not cause defective receptor localization or surface trafficking (Erbs et al., 2014). Importantly, MOP-mcherry retained unchanged receptor density as well as [D-Ala^2^, N-MePhe^4^, Gly-ol]-enkephalin (DAMGO) binding and efficacy and agonist-induced internalization compared to MOP. Moreover, behavioral effects of morphine in knock-in mice were similar to wild type animals: acute and chronic thermal analgesia, physical dependence, sensitization and rewarding properties revealed no significant differences with wild type animals (Erbs et al., 2014). These data suggest that predominant intracellular localization of MOP-mcherry receptors with low expression at the cell surface indeed reflect endogenous wild type receptor subcellular distribution under basal conditions, as observed in enteric neurons (Poole et al., 2011). In addition, internalization kinetics of MOP-mcherry upon activation by the agonist DAMGO in hippocampal primary neuronal cultures (Erbs et al., 2014) were similar to those reported for DAMGO promoted internalization of endogenous wild type receptors in the rat spinal cord (Trafton et al., 2000) and in organotypic cultures of guinea pig ileum (Minnis et al., 2003) or to Fluoro-dermorphin-induced sequestration in rat cortical primary neurons (Lee et al., 2002). This supports once again the use of fluorescent knock-in mice to study endogenous receptor trafficking. Of note, DAMGO promotes Flag-MOP receptor internalization with similar kinetics in transfected striatal primary neurons (Haberstock-Debic et al., 2005), in adenovirus infected primary cultures from DRG (Walwyn et al., 2006) or in neurons of the locus coeruleus in brain slices from transgenic FLAG-MOP receptor mice (Arttamangkul and Quillinan, 2008).

## CONCLUSIONS AND IMPACT FOR DRUG DESIGN

Fluorescent knock-in mice represent a substantial technical improvement in basic science. Precise identification and localization of the neurons expressing the GPCR of interest and reliable monitoring of receptor subcellular localization are both essential in understanding the physiopathological roles of endogenous GPCRs. This was greatly anticipated, given the difficulties encountered by many on the grounds of poor specificity of the available antibodies for GPCR targeting. The main surprising finding is maybe that the presence of the FP at the *C*-terminus of the GPCR does not significantly alter the behavioral output: this observation fully validates the technology. However, fluorescent knock-in animals available to date target a handful of class A GPCRs only. The potency of the model being now clearly established, one would expect rapid expansion to other receptors, in particular those with critical roles in human pathologies. Forefront candidates include class C GABA_B_ and metabotropic glutamate receptors, both of which are involved in a wide range of neurological disorders such as schizophrenia, neuropathic pain, cerebral ischemia, mood disorders and substance abuse (Benes and Berretta, 2001; Delille et al., 2013; Kumar et al., 2013). Fluorescent knock-in animals would enable to revisit heterodimerization mechanisms, membrane targeting and cellular distribution patterns of these obligatory heterodimers *in vivo*. Furthermore, the relation between multimer scaffold composition, in particular GABA_B_ auxiliary subunits, and neuronal or synaptic functions could also be readily examined to refine our current understanding of the variations in pharmacological and functional responses mediated by native receptors (Gassmann and Bettler, 2012).

The knock-in mice bearing GPCR-FP fusions already contributed to understanding the fundamental concepts of distinct signaling or regulatory responses recruited by different agonists of the same GPCR. These essential aspects of biased agonism are a growing central concern in drug discovery in the hope of developing strategies that ally high efficacy with low or no side effects. In addition, GPCR-FP fusions could bring considerable knowledge regarding functional aspects of receptor activity and internalization to evaluate the therapeutic potency of allosteric modulators. This very active field of research is mainly targeting class C GPCRs with well identified allosteric and orthosteric binding sites such as metabotropic glutamate or GABA_B_ receptors but relevance for class A GPCRs is attracting increasing attention (Nickols and Conn, 2014). Direct visualization of the neurons of interest, either by FP under the control of a GPCR promoter or by expression of the GPCR fluorescent construct, also represents a significant breakthrough by making subsequent targeted investigations available. This includes electrophysiological recordings on previously identified cell, cell isolation by fluorescence-activated cell sorting for further biochemical (Western Blotting) and molecular (RT-PCR) downstream analysis or highly specific and efficient immunoprecipitation of the interacting partners. The presence of the FP also gives access to imaging techniques with which receptor population tracking within membranes can be achieved, by fluorescence recovery after photobleaching or fluorescence resonance energy transfer. The latter also opens ways to identify heteromer formation between GPCRs or between a GPCR and a ligand-gated channel and to investigate *in vivo* their intracellular fate and impact on signaling cascades. All these technological developments will undeniably contribute to deepening our current knowledge of GPCR controlled molecular and cellular processes and ultimately will benefit to drug design and screening.

## Conflict of Interest Statement

The authors declare that the research was conducted in the absence of any commercial or financial relationships that could be construed as a potential conflict of interest.
